# Intracranial Hemorrhage in a Patient With COVID-19: Possible Explanations and Considerations

**DOI:** 10.7759/cureus.10159

**Published:** 2020-08-31

**Authors:** Bhanu Gogia, Xiang Fang, Prashant Rai

**Affiliations:** 1 Neurology, University of Texas Medical Branch, Galveston, USA

**Keywords:** covid-19 and ich, intracranial hemorrhage and sars-cov-2, covid-19 neurological outcomes, fatal outcomes covid-19, stroke and covid-19

## Abstract

Some of the reported neurological manifestations of COVID-19 are encephalopathy, headache, ischemic, hemorrhagic stroke, Miller Fisher syndrome, cranial neuropathies, and Guillain-Barre syndrome. We report a case of a 75-year-old COVID-19 patient with life-threatening intracranial hemorrhage. The initial labs on admission showed D-dimer of 1.04 µg/mL, which increased to 3.74 µg/mL the next day, PT/INR of 13.7 seconds/1.2, aPTT of 22 seconds, fibrinogen of 386 mg/dL, WBC of 9.71 K/µL, Hgb of 14.1 g/dL, platelet of 315 x 10^3^/µL, LDH of 965 U/L, and CRP of 35.2 mg/dL. In addition to aspirin and Plavix (clopidogrel), the patient was started on a therapeutic dose of enoxaparin due to elevated D-dimer. A few days later, the patient had a change in the neurological examination. The CT of the head without contrast revealed a left-sided acute subdural hematoma, causing left to right midline shift, a large left temporal intraparenchymal, and subarachnoid hemorrhage with transtentorial herniation, leading to death. This case illustrates a combination of factors including hypertension, triple therapy (aspirin, clopidogrel, and enoxaparin), and underlying coagulopathy due to COVID-19, which contributed to the life-threatening intracranial hemorrhage in this patient. Therefore, this raises a concern about the safety of starting these patients preemptively on a therapeutic dose of anticoagulation.

## Introduction

The coronavirus disease 2019 (COVID-19) pandemic caused by severe acute respiratory syndrome coronavirus 2 (SARS-CoV-2) has spread globally. As of June 20, 2020, there were 8,546,919 cases and 456,726 deaths across 216 countries [1]. The numbers continue to increase, causing a marked impact on healthcare and economy worldwide. At the beginning of the pandemic, COVID-19 was primarily considered to be respiratory, and neurological complications were considered to be relatively rare. But there is growing evidence suggesting neurological involvement in COVID-19 patients. A study from Wuhan reported neurological involvement in around 36.4% of the patients, whereas another study from Spain reported neurological symptoms in 57.4% of the patients [2-3]. Although it is difficult to establish causality at this time, the possible mechanisms known thus far are, first, neurotropism toward angiotensinogen-converting enzyme (ACE2) receptors present in the central nervous system (CNS), second, autoimmunity, and, third, cytokine storm and hypercoagulable state [4-7]. Some of the reported neurological manifestations involving the CNS and peripheral nervous system (PNS) are encephalopathy, headache, ischemic, hemorrhagic stroke, transient ischemic attack, Miller Fisher syndrome, cranial neuropathies, and Guillain-Barre syndrome [8-10]. Per the existing literature, neurological complications occur more commonly in people with older age, diabetes, hypertension, and cardiovascular diseases [11]. Some of the laboratory markers that are reported to be deranged in COVID-19 patients with CNS involvement are elevated IL-6 (interleukin-6), procalcitonin, D-dimer, C-reactive protein (CRP), increased blood urea nitrogen, decreased lymphocytes, and decreased platelets compared to the patients without CNS manifestations. Cerebrospinal fluid (CSF) analysis in patients with Miller Fisher syndrome showed elevated protein, whereas, in other patients, CSF has reportedly been normal [8]. Autopsy results of these patients demonstrated hyperemic, edematous brain tissue. Only a handful of intracranial hemorrhage cases in COVID-19 patients are there in the literature [12-13]. We hereby report a unique case of a COVID-19 patient with severe illness who developed subdural hematoma (SDH), subarachnoid hemorrhage (SAH), and intraparenchymal hemorrhage (IPH), causing midline shift and herniation syndrome, leading to death. With the help of this case, the authors have highlighted some considerations prior to starting anticoagulation in COVID-19 patients.

## Case presentation

**Figure 1:**
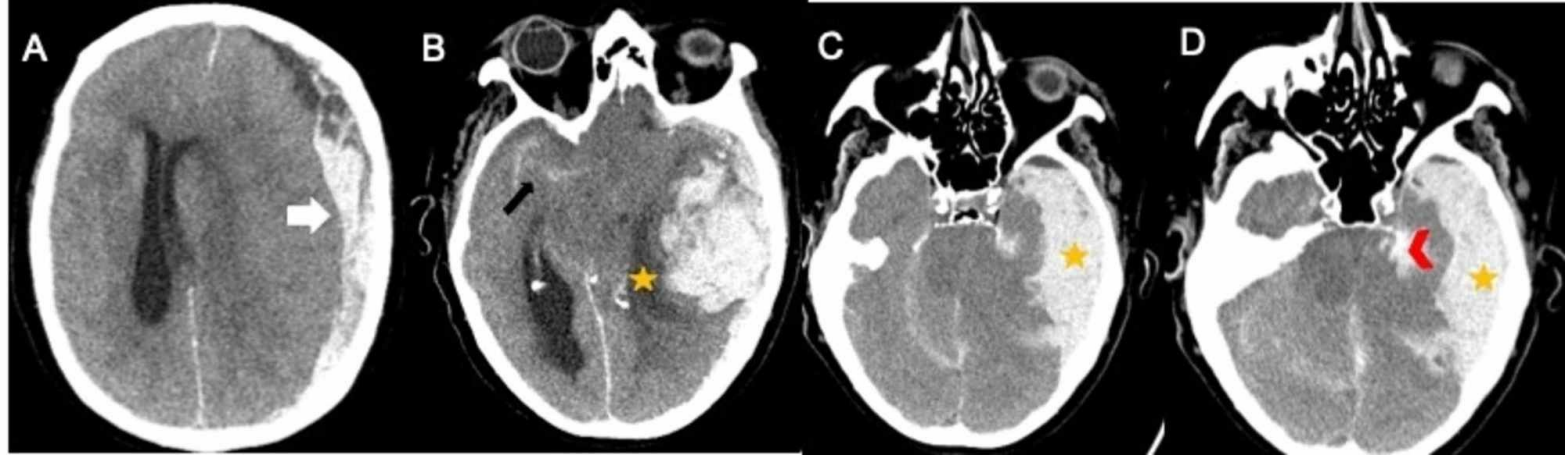
CT of the head without contrast (image A) shows crescent-shaped hyperdensity (white arrow in image A) on the left frontoparietal cortex with midline shift suggesting hyperacute and active hemorrhage. Images B-D show hyperdensity in the right Sylvian fissure consistent with subarachnoid hemorrhage (black arrow in image B) and intraparenchymal hematoma in the left temporal lobe with surrounding hypodensity (yellow star in images C and D) causing severe transtentorial herniation (red arrow in image D).

The patient is a 75-year-old female with a past medical history of hypertension, hyperlipidemia, hypothyroidism, peripheral artery disease status post two stents (on aspirin and clopidogrel), and 50 pack-year smoking history. The patient presented to the hospital institution with acute hypoxia secondary to COVID-19. Initial laboratory results on admission showed elevated D-dimer of 1.04 µg/mL (reference range: less than 0.50 µg/mL FEU), which increased to 3.74 µg/mL the next day. Some of the other laboratory findings were slightly elevated: prothrombin time/international normalized ratio (PT/INR) of 13.7 seconds/1.2 (reference range for PT/INR: 10.1-12.6 seconds/<1.1), normal activated partial thromboplastin time (aPTT) of 22 seconds (reference range: 26-36 seconds), normal fibrinogen of 386 mg/dL (reference range: 167-453 mg/dL), white blood cells (WBC) of 9.71 K/µL (normal range: 4-11 K/µL), hemoglobin (Hgb) of 14.1 g/dL (normal range: 11.6-15.0 g/dL), platelets of 315 x 10^3^/µL (normal range: 166-358 x 10^3^/µL), elevated lactate dehydrogenase (LDH) at 965 U/L (reference range: 300-600 U/L), and elevated C-reactive protein (CRP) at 35.2 mg/dL (reference range: <0.8 mg/dL). The patient was admitted to the medical intensive care unit due to hypoxia and respiratory distress and was started on remdesivir. Over the course of admission, she had worsening respiratory distress and was intubated five days later. The patient was started on a prophylactic dose of enoxaparin. Infectious workup was significant for blood cultures growing bacillus species and urinary culture growing *Escherichia coli* for which she received vancomycin and ceftriaxone. Transthoracic echocardiogram did not show any vegetations. Due to elevated D-dimer the following day, enoxaparin was changed to a therapeutic dose in addition to aspirin and clopidogrel. The patient self-extubated herself but was successfully reintubated the same day. Over the course of the next five days, the patient was weaned off vasopressors and started on midodrine. Seventeen days post-admission, the patient was found to be unresponsive to pain stimuli with pupils dilated and not reactive to light. This led to the activation of stroke code. Neurological exam revealed no response to pain, pupils 6 mm fixed and dilated bilaterally, negative oculocephalic, negative corneal reflexes, cold caloric, cough, gag, and spontaneous breathing. There were some spikes noted in her blood pressure over 72 hours, with readings as high as 198/83 mmHg and as low as 77/47 mmHg. The same day, lab values did not show any worsening findings to suggest any underlying obvious coagulopathy and were as follows: WBC of 8.03/µL, Hgb of 8 g/dL, platelets of 201 x 10^3^/µL, PT of 11.8 seconds, INR of 1, and aPTT of 30 seconds, LDH down trended to 608 U/L, and CRP trended down to 2.6 mg/dL. Computed tomography (CT) of the head without contrast (Figure 1D) showed large left temporal IPH, SAH, and left-sided SDH (acute/hyperacute), causing left to right midline shift and compression of the brain stem. The patient was not amenable for any neurosurgical intervention and was declared brain dead. Shortly after palliative extubation, the patient passed away, and with the consent of the family, autopsy was performed. The autopsy reported a left-sided IPH in the temporal lobe with about 100 mL of blood in the subdural space. Bilateral lungs showed diffuse consolidation with alternating pale areas with hemorrhages (possible diffuse alveolar damage; weights: left, 820 gm; right, 870 gm). Bilateral pleural spaces contained serosanguinous effusions (left: 300 mL, right: 250 mL).

## Discussion

With the increasing number of COVID-19 cases around the world, more and more cases with neurological manifestations are being unfolded. Prior to this, there are only a small number of cases reported with SAH and IPH, including the study from Wuhan [2,12-13]. This is the first case of severe COVID-19, with SDH, SAH, and IPH leading to severe herniation and death. COVID-19 is known to be a prothrombotic state [4], and severity is linked to the elevated D-dimer levels [9]. At presentation, the coagulation panel including PT/INR and aPTT were normal except D-dimer, which was slightly elevated at 1.04 µg/mL and increased to 3.74 µg/mL the next day. During the hospitalization, with improving D-dimer (0.82), fibrinogen, LDH, and normal platelets, there was no evidence of any imminent disseminated intravascular coagulation. The current literature on the use of anticoagulants and continuing antiplatelets in COVID-19 patients is ambiguous. The decision to start anticoagulation is individualized based on the severity of symptoms and the coagulation profile of these patients, which includes D-dimer, platelets, fibrinogen levels, PT/INR, aPTT, CRP, and LDH [14]. While some have suggested not to use a full-dose anticoagulant unless clinically indicated in severe cases [14], others have suggested that the use of systemic anticoagulation might be associated with improved outcomes in these patients [15]. The patient described in this report had normal fibrinogen, INR, and platelet count. Her CRP and LDH also trended down. She was on aspirin and clopidogrel prior to admission and was started on therapeutic enoxaparin. However, the presence of SDH, SAH, IPH of different ages [16], and serosanguinous effusions in pleural spaces on autopsy favored underlying coagulopathy. Some of the risk factors associated with higher mortality are age and perhaps being on dual antiplatelet therapy for peripheral vascular disease. A retrospective study reported that hypertension, diabetes, coronary artery disease, and hypercholesterolemia did not pose any significant risk on the mortality when compared to non-COVID-19 patients [9]. The same study showed that COVID-19 patients with a cerebrovascular injury who underwent acute phase therapies (intravenous thrombolysis and endovascular therapies) suffered the worst outcomes when compared to those who did not [9]. This could be explained due to the increased propensity of hemorrhagic events due to underlying coagulopathy. The ICH described in Figures 1A-1D is somewhat atypical for a purely hypertensive hemorrhage, which typically is intraparenchymal and occurs commonly in the basal ganglia, cerebellum, pons, or thalamus. From this case, it can be postulated that hypertension and triple therapy contributed to the development of ICH in this patient with underlying COVID-associated coagulopathy. This case highlights that the decision to start anticoagulation should be weighed along with the indication to continue antiplatelet therapy. If triple therapy is warranted in such patients, perhaps a baseline CT of the head followed by an aggressive blood pressure control should be done.

A limitation of this study is the lack of prior head imaging as the patient did not have any neurological concerns prior to the decline.

## Conclusions

Overall, the combination of hypertension, triple therapy (aspirin, clopidogrel, and enoxaparin), and underlying coagulopathy due to COVID contributed to the life-threatening intracranial hemorrhage in this patient. With COVID-19 being a prothrombotic state, the question remains about the safety of starting these patients preemptively on a therapeutic dose of anticoagulation without any evidence of an acute thrombotic event. Perhaps, if anticoagulation is warranted, then antiplatelet therapy should be discontinued to prevent such life-threatening bleeding complications.
